# Influence of Resorcinol to Sodium Carbonate Ratio on Carbon Xerogel Properties for Aluminium Ion Battery

**DOI:** 10.3390/ma15072597

**Published:** 2022-04-01

**Authors:** Martin Eckert, Heena Suthar, Jean-Francois Drillet

**Affiliations:** DECHEMA-Forschungsinstitut, Theodor-Heuss-Allee 25, 60486 Frankfurt am Main, Germany; martin.eckert@dechema.de (M.E.); heena.suthar@dechema.de (H.S.)

**Keywords:** carbon, xerogel, soft-template, resorcinol, aluminium-ion battery, pseudo-capacitance, intercalation, X-ray diffraction, conductivity, Raman

## Abstract

Carbon xerogels were synthesized using a soft-template route with resorcinol as the carbon source and sodium carbonate as the catalyst. The influence of the resorcinol to catalyst ratio in the range of 500–20,000 on pore structure, graphitic domains, and electronic conductivity of as-prepared carbon xerogels, as well as their performance in an aluminium ion battery (AIB), was investigated. After carbonization steps of the polymers up to 800 °C, all carbon samples exhibited similar specific volumes of micropores (0.7–0.8 cm³ g^−1^), while samples obtained from mixtures with R/C ratios lower than 2000 led to carbon xerogels with significantly higher mesopore diameters up to 6 nm. The best results, in terms of specific surface (1000 m² g^−1^), average pore size (6 nm) and reversible capacity in AIB cell (28 mAh g^−1^ @ 0.1 A g^−1^), were obtained with a carbon xerogel sample synthetized at a resorcinol to catalyst ratio of R/C = 500 (CXG_500_). Though cyclic voltammograms of carbon xerogel samples did not exhibit any sharp peaks in the applied potential window, the presence of both oxidation and a quite wide reduction peak in CXG_500–2000_ cyclic voltammograms indicated pseudocapacitance behaviour induced by diffusion-controlled intercalation/de-intercalation of AlCl_4_^−^ ions into/from the carbon xerogel matrix. This was confirmed by shifting of the (002) peak towards lower 2θ angle values in the XRD pattern of the CXG_500_ electrode after the charging step in AIB, whereas the contribution of pseudocapacitance, calculated from half-cell measurements, was limited to only 6% of overall capacitance.

## 1. Introduction

The demand for electrically rechargeable energy storage systems has increased significantly over the last few years. Apart from classical systems, such as lead-acid and nickel-metal hydride (NiMH) systems, a huge number of established and merged application devices rely on lithium ion batteries (LIB) due to their impressive cell performance in terms of cell voltage (3–3.6 V), energy density (220 Wh kg^−1^/450 Wh L^−1^) and long-term cycling ability [1]. Because of the enormous demand for LIBs and the consequential rise in lithium price, as well as for critical materials such as cobalt, shortage in raw materials within the next decades appears to be inevitable [2,3]. Therefore, there is an urgent need to develop novel battery systems that rely on highly abundant, non-critical metals, such as zinc, sodium, magnesium or aluminium. One of the most promising candidates is the Al-ion battery (AIB) that typically consists of highly graphitic carbon (natural graphite [4,5,6,7,8,9,10,11,12], graphene [13,14,15,16,17], graphene oxide [18] or carbon foams [11]) as the positive electrode for Al-ion intercalation, an AlCl_3_ containing ionic liquid, such as 1-ethyl-3-methylimidazoliumchloride (EMimCl), as the aprotic electrolyte, and an aluminium metal foil as the negative electrode for electrochemical Al deposition/stripping. The main benefits of AIB are related to the use of highly abundant and cheap graphite (1.3 $ kg^−1^) and aluminium (2 $ kg^−1^) materials [19,20], extraordinarily high power densities [21] (up to 90 kW kg^−1^_Carbon_), extremely high cycle stability (up to half a million cycles) [6] and highly efficient Al recycling infrastructure. The main drawbacks and technical challenges to overcome are related to the highly water-sensitive and corrosive properties of the EMimCl/AlCl_3_ electrolyte, aluminium dendrite growth within the glass fibre separator that acts as an insulator and electrolyte reservoir, as well as graphite delamination issues over cycling. During the AIB charging step, negatively charged aluminium tetrachloride ions (AlCl_4_^−^) intercalate [22] into the graphite matrix, while, at the same time, aluminium heptachloride (Al_2_Cl_7_^−^) species are electrochemically reduced to Al^0^ on the aluminium foil according to the following reactions:

$$C_{n}+{\mathrm{AlCl}}_{4}^{-}\overset{\mathrm{Intercalation}}{\to }C_{n}\left[{\mathrm{AlCl}}_{4}\right]+e^{-}$$

$$4{\;\mathrm{Al}}_{2}{\mathrm{Cl}}_{7}^{-}+3{\;e}^{-}\overset{\mathrm{Deposition}}{\to }\mathrm{Al}+7{\;\mathrm{AlCl}}_{4}^{-}$$

Due to the large size of the intercalating anion (5.4 Å for AlCl_4_^−^ vs. 1.34 Å for Li^+^) [23], the graphene layers (distance between two graphene layers: ~3.3 Å) at a certain point undergo irreversible expansion that can led to delamination of the active layer material [11,24]. To overcome this issue, three-dimensional carbon structures, such as foams, were developed [11]. Due to their open-pore structure, volume expansion/contraction during successive charging/discharging steps can be minimized. Since commercially available carbon products rarely fulfil further prerequisite properties, such as high corrosion resistance and electronic conductivity that allow both fast reversible intercalation/deintercalation of large multivalent ions as well as optimal mass transport, much effort has been invested in designing new carbon structures by hard-template and soft-template routes [25]. Galeano et al. [26] reported the synthesis of mesoporous carbon hollow spheres via a hard-template route, in which divinylbenzene as the carbon precursor and iron (III) nitrate as the catalyst were introduced into the pores of silica spheres or colloidal crystals by impregnation, followed by carbonization and the time-consuming removal of silica using a hot alkali or hydrofluoric acid solution. Importantly, the pore size and morphology of mesoporous carbon network (MCN) replicas are difficult to tune due to the limited choice of appropriate hard-template materials [27], such as polymer microspheres, porous membranes, plastic foam, ion exchange resin, carbon fibre and porous anodic aluminium oxide [28].

Therefore, soft-template routes appear to be more suitable for designing carbon materials with tuneable properties. A broad overview of soft-templating routes is provided by Chuenchom et al. [29]. Commonly, mesostructured carbons are obtained via an organic-organic self-assembly process induced by hydrogen bonding between a carbon precursor (e.g., phenolic-formaldehyde resin) and a template (e.g., amphiphilic triblock copolymer Pluronic F127 (EO_106_PO_70_EO_106_)). Using this kind of soft-template material, mesoporous carbons can be produced in the form of films, monoliths or particles of micrometre size [30].

Carbon gels are synthetic polymeric carbon materials known for their tuneable porosity, surface area and morphology. Aerogels, cryogels and xerogels are three organic gels that are synthesized by a sol-gel polycondensation method and differ from each other regarding drying procedure [31]. While aerogels are produced by a drying procedure with supercritical CO_2_ after solvent exchange (acetone or ethanol), cryogels are obtained by freeze-drying in which the solvent is removed by sublimation under low pressure. However, these two latter drying procedures are expensive and difficult to handle. In contrast, carbon xerogels (CXG) are produced by successive simple vacuum drying and resorcinol-formaldehyde pyrolysis steps. In the last decade, the feasibility of use of carbon xerogels as material for supercapacitors has been intensively investigated [32,33,34,35], as well as for anode material in Li-ion batteries [36,37,38].

In this study, the influence of catalyst concentration during the polycondensation of resorcinol with formaldehyde on carbon xerogel morphology was systematically studied for different resorcinol to catalyst (R/C) ratios.

## 2. Materials and Methods

### 2.1. Synthesis of Xerogels

**Figure 1:**
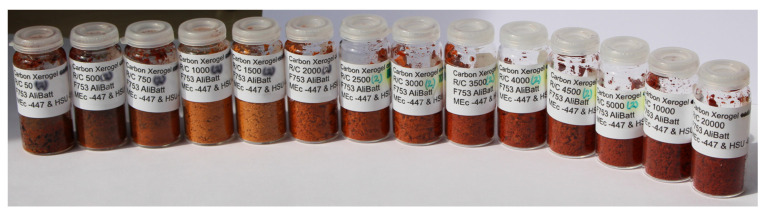
As-prepared resorcinol-formaldehyde resins after drying step from high catalyst concentration (R/C = 50) on the left side to low concentration (R/C = 20,000) on the right side.

Fourteen different xerogel samples were prepared using resorcinol (R) to sodium carbonate as catalyst (C) ratios in the range of R/C = 50–20,000 and keeping resorcinol to formaldehyde at a molar ratio of 0.5. First, 1.061 g of sodium-carbonate (Na_2_CO_3_) (>99.99%, Sigma Aldrich, Darmstadt, Germany) was poured into ultrapure water (18.2 MΩ, ELGA Prelab^®^, Celle, Germany) to obtain a standard solution of 0.1 M Na_2_CO_3_. Then, 1.0 g of resorcinol (>99%, Sigma Aldrich, Darmstadt, Germany) was dissolved in 1.95 mL of Na_2_CO_3_ catalyst solution for a given R/C molar ratio under stirring conditions. After complete dissolution, 1.355 mL of 30% formaldehyde solution (35 Vol%, Sigma Aldrich, Darmstadt, Germany) was added resulting in a molar R/F ratio of 0.5. The polymerization reaction was carried out in a water bath at 65 °C for 72 h. To remove residual water, a drying step was conducted at 125 °C and 0.1 mbar in a self-build Schlenk-line apparatus. The heating step led to a change in colour of the as-prepared polymers (see Figure 1).

Finally, carbonization of the samples was performed in a tubular oven (ROK 3/30, W. C. Heraeus, Hanau, Germany) under N_2_ atmosphere by applying two successive heating steps at 400 and 800 °C for 2h each and using a temperature ramp of 5 K min^−1^. The yielded carbons showed differences in density where the highest and lowest values were observed at CXG_50_ and CXG_20,000_, respectively (see values in Table A1). At CXG_50_ especially, very hard and dense particles were formed. One possible explanation might be the higher concentration of sodium carbonate in the range of R/C = 50–1500 that favoured fast formation of large aggregates [39]. Immediately after adding formaldehyde to the R/C_50_ solution, the colour started to change from clear white to red/orange. After 2 h, the polymerization step was completed, resulting in a dark red colour polymer. For R/C ratios in the range of 500–1500, the whole polymerization was accomplished after 18–24 h. For R/C > 1500 samples, the polymerization step took 35–45 h due to a slow reaction rate. It seems that a slow polymerization reaction favoured the formation of small aggregates which resulted after carbonization in less dense carbon particles. At very low Na_2_CO_3_ concentrations, such as those used during R/C_10,000_ and R/C_20,000_ synthesis mixtures, the solution was colourless even after 60 h reaction time.

### 2.2. Preparation of Cathode Material for AIB Experiments

Five selected carbonized xerogels (CXG) were ball-milled in ethanol at 400 rpm for 2 h (PM 100, Retsch, Haan, Germany). Then, an appropriate amount of 90 wt% CXG powder and 10 wt% polytetrafluoroethylene (PTFE, 30 wt% from Quintech, Göppingen, Germany) were mixed in 70 Vol% isopropanol (VWR, Darmstadt, Germany)/water (ELGA Purelab^®^, Celle, Germany) by an Ultra-Turrax^®^ (T-18, IKA, Staufen, Germany) dispersing instrument. The resulting suspension was spray-coated on a glass fibre-separator (0.25 mm in thickness, GF/A Whatman™/Cytiva, Freiburg, Germany) until a loading of the cathodes of 2–3 mg_CXG_ cm^−2^ was achieved. As-prepared cathodes were cut in 10 mm discs and dried at 125 °C for at least 12 h to remove all residual water. Electrodes with commercial graphite (natural high-conductive graphite, Prographit, Untergriesbach, Germany) were prepared analogous to CXG electrodes, however, without ball-milling-step.

### 2.3. Electrochemical Measurements

For half-cell measurements, PTFE T-Cells (Ø = 10 mm, Swagelok-type, EM-Technik, Maxdorf, Germany) were assembled in a glovebox (UniLab Pro, H_2_O/O_2_ < 0.1 ppm, MBraun, Garching, Germany) by successively stacking a 10 mm glassy carbon (GC) rod (SIGRADUR^®^, HTW, Thierhaupten, Germany), a glass fibre-coated carbon xerogel (~2 mg loading) working electrode, two GF/D separator layers (670 µm thickness, Whatman™/Cytiva, Freiburg, Germany) impregnated with 350 µL electrolyte (n(EmimCl):n(AlCl_3_) = 1:1.5, IoLiTec, Heilbronn, Germany) and an Al-rod (Ø = 10 mm, 99.999%, HMW Hauner, Röttenbach, Germany) as counter electrode. Finally, an Al-rod (Ø = 6 mm, 99.999%, HMW Hauner, Röttenbach, Germany) was placed in the perpendicular tube of the T-cell that acted as the pseudo-reference electrode. Cyclovoltammetry measurements were carried out in a glovebox with a potentiostat/galvanostat (Im6EX, Zahner, Kronach, Germany) at 1 mV s^−1^ scan rate. (Pseudo)capacitance and Faradaic contributions were determined by scan-rate variation of cyclic voltammograms according to Dunn’s method [40]. Charge/discharge and current/voltage measurements of AIB cell were performed in PTFE straight cells (Ø = 10 mm, Swagelok-type, EM-Technik, Maxdorf, Germany) with a battery tester (BCS810, Biologic, Seysinet-Pariset, France). The activation procedure of all AIB cells consisted of 25 cycles at 0.1 A g^−1^. Afterwards, the performance of AIB cells was investigated at 0.1–5.0 A g^−1^ current density for 10 cycles with a lower voltage limit of 0.4 V during discharge for all cells and an upper voltage limit of 2.3 V during charge for cells with CXG_500_ and natural graphite, and 2.4 V for cells with CXG_750–2000_.

### 2.4. Instrumental Analysis

XRD and Raman analysis of carbon powder samples was carried out in a diffractometer (D8 Advance, BRUKER, Karlsruhe, Germany) with CuKα radiation of 1.541874 Å and Raman microscope (inVia, Renishaw, Pliezhausen, Germany) at 532 nm, respectively. The intercalation of chloroaluminate into graphite or CXG matrix was studied by ex situ XRD analysis. For this purpose, a charged graphite or CXG electrode was placed in an air-tight BRUKER™ specimen holder. The air tightness required to be assured because of the strong hydrolysis properties of AlCl_3_^−^-containing ionic liquid electrolyte. SEM images were acquired with a field emission microscope (SU5000, Hitachi, Krefeld, Germany). Conductivity measurements were performed in a pressurized device designed by ZBT (Zentrum für Brennstoffzellentechnik, Duisburg, Germany). Physisorption measurements of carbon powder materials were conducted with argon at 87 K (Autosorb iQ Station, Quantachrome / 3P Instruments, Odelzhausen, Germany). The whole mass-normalized surface area A_sum_ and pore volume V_sum_ were calculated by applying DFT-fitting of cylindrical and spherical pores to measured adsorption isotherms (QSDFT-fit).

## 3. Results

### 3.1. Physisorption of CXGs

**Figure 2:**
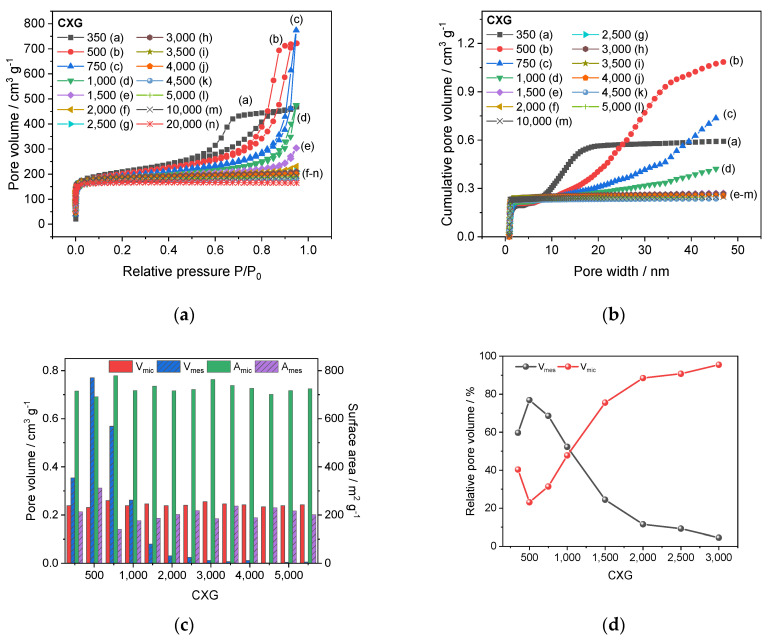
Characterisation of CXG by isotherms (**a**), pore size distribution with respect to V_sum_ (**b**), contribution of microporous and mesoporous pore volume and surface area (**c**) and repartition of relative mesopore and micropore volume (**d**).

Plots of samples produced within 350 (a) < R/C < 2000 (b) showed a typical hysteresis for type IV isotherms indicating the presence of mesopores (Figure 2a). For R/C > 2500, named (f–n), the isotherm of carbons shifted to type 1a which indicated nearly 100% microporous textures. This was confirmed by an average pore size distribution of 6.0 nm for CXG_500_ and only 1.7 nm for CXG samples with R/C > 2000. The analysis of cumulative pore volume V_sum_ in relation to R/C showed that V_sum_ subsequently decreased with R/C ratio from 1.0 cm^3^ g^−1^ in CXG_500_ (a) to 0.5 cm^3^ g^−1^ in CXG_1000_ (d) and then amounted to 0.2–0.3 cm^3^ g^−1^ for R/C > 1500 (e–m), see Figure 2b. For the as-prepared carbon xerogels samples, the contribution of macropores was excluded. The micropore volume V_mic_ and surface area A_mic_ were calculated according to the Dubinin–Radushkevich (DR) equation [41,42,43,44]. The mesopore volume V_mes_ and surface area A_mes_ were consequently calculated by subtracting V_sum_ with DR-derived pore volumes V_mic_ and surface area A_mic_, respectively.

The mass-normalized surface area A_sum_ was determined to be around 926 m^2^ g^−1^ on average with a maximum of 1004 m^2^ g^−1^ for CXG_500_ and a minimum of 894 m^2^ g^−1^ for CXG_1000_. The surface area of all CXG_500–10,000_ samples were higher than some values reported in other investigations (300–700 m^2^ g^−1^) [34,41,42,45]. Moreover, the overall pore volume V_sum_ of 0.2–1.0 cm^3^ g^−1^ was similar to values referred to in the literature [34,41,42].

As shown in the bar chart represented in Figure 2c, the specific volume of micropores V_mic_ was quite similar for all CXG samples and fluctuated between 0.23–0.26 cm^3^ g^−1^ which was in good accordance with published values [41,42]. As expected from the isotherm shapes, the mesopore volume V_mes_ culminated in the CXG_500_ sample at 0.77 cm^3^ g^−1^ and decreased rapidly to 0.08 cm^3^ g^−1^ in CXG_1500_. Surprisingly, the specific surface area A_mes_ of the carbon samples did not fluctuate very much (312–141 m^2^ g^−1^). A detailed overview of the textural properties is given in Appendix A Table A2.

The contribution of V_mes_ and V_mic_ in selected CXG samples is shown in Figure 2d. We found that the pore volume of CXG_500_ consisted of 85% V_mes_ and 15% V_mic_. For CXG_750_, 80% V_mes_ were determined. With higher R/C values, the amount of V_mes_ dropped to 21% in CXG_2000_ while the amount of V_mic_ increased up to 79%.

Since carbon materials with mesoporous domains are usually preferred in electrochemical systems due to their ability to enhance better diffusion of electrolyte species to active sites and consequently overall capacity, only CXG_500–2500_ samples were considered further for investigation in AIB laboratory cells.

### 3.2. X-ray Diffraction

**Figure 3:**
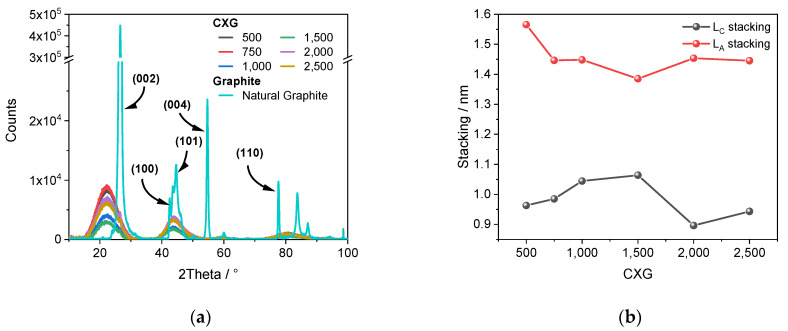
XRD diffractogram of selected CXG and natural graphite (**a**). Stacking width L_A_ and stacking height L_C_ of graphene crystallites as a function of R/C ratio during CXG synthesis (**b**).

XRD diffractograms of CXG (see Figure 3a) showed three dominant broad peaks at 2θ = 22 ± 1°, 43 ± 1° and 80 ± 1°. Broad peaks are typical for amorphous carbons [46,47] and are a mixture of sp^2^ and sp^3^ hybridized carbons [48] with a turbostratic arrangement. For comparison, a pure natural graphite (natural high conductive graphite, Prographit) is also shown with a clear visible crystal-plane pattern (green pattern). Since it is difficult to correctly interpret the crystal structure of amorphous carbons, the peaks with (hkl) nomenclature should be carefully assigned. Here we followed Maldonado-Hódar et al. [46] and assigned peaks at 2θ = 22° and 43° to (002) and (101) planes, respectively. The intense peak at 80° in the CXG diffractogram could not be definitively linked to a specific crystal plane.

From the (002) peak, the layer interspacing value (d_002_) was determined to be 3.335 Å for the natural graphite powder, a value comparable to those reported for different graphite materials (3.35–3.73 Å) in [4,8,49]. The diffractograms of CXG_500–2500_ were very similar to each other with small differences of several hundred counts which indicated a similar structure. The calculated d_002_ values for all CXG samples varied between 3.6–3.8 Å, values which were comparable to those extracted from plots of pyrolyzed resorcinol-formaldehyde resins (3.4–4.1 Å) [46,50,51,52,53]. Interestingly, the interspacing values of as-prepared CXG were 8–14% higher than those of natural graphite which might be advantageous for the intercalation of large AlCl_4_^−^ anions.

The (002) peak was induced by the stacking height (L_C_) of crystallite, while the (101) peak was induced by the stacking width (L_A_). From lithium-ion systems, it is well-known that there is a large difference in the electrochemical activity of basal (lateral size, L_A_) and edge surfaces (stacking height, L_C_). Chung et al. [53], as well as Zaghib et al. [54], showed that the basal plane area contributes around 7× less to electrochemical activity than the edge sites which are important for the intercalation process. L_C_ and L_A_ values of different carbon samples were calculated by applying the Scherrer equation (Equation (1)) for (002) and (101) reflexes with L = stack height/width, K = Scherrer factor, λ = wavelength X-ray source, ∆(2θ) = FWHM (full-width at half maximum) of (hkl) reflex, and θ_0_ the Bragg-reflex. FWHM values of CXG_s_ were calculated by Gaussian peak deconvolution in Origin^®^ 2020. Graphitic carbons can have L_A_ values >> 100 nm [54], while non-graphitic carbons (NCG) have very small particles in the low nanometre-range [48,55].

(1) $$L=\frac{K\cdot \lambda }{\Delta \left(2\theta \right)\cdot {\cos\theta }_{0}}$$

L_C_ stacking (Figure 3b) increased from 0.96 nm for CXG_500_ to 1.06 nm for the CXG_1500_ sample. The corresponding L_A_ values dropped from 1.57 nm in CXG_500_ to 1.39 nm in CXG_1500_. For CXG_2000_ and CXG_2500_, the calculated L_A_ value was 1.45 nm. The L_C_ value of CXG_2000_ had a minimum height of 0.90 nm, whereas CXG_2500_ showed a slightly higher stacking height of 0.94 nm. The maximum L_A_ stacking was observed in CXG_500_ with 1.57 nm, while the maximum L_C_ stacking for CXG_1500_ was measured as 1.06 nm. The average crystallite size in width and height was determined to be 1–2 nm, which is typical for resin-based NCGs as reported by Pfaff et al. [55]. Overall, it can be concluded that the resorcinol to carbonate ratio had almost no influence on the carbonized resins.

For comparison, the L_C_ and L_A_ values of crystallite in natural graphite with well-defined crystal plane patterns were calculated to be 66.3 ± 0.5 nm and 30.2 ± 0.9 nm, respectively. The overall measured intensity of the (002) plane was not only two magnitudes higher than for CXG, but also much higher than that of (101) planes, which is an indication of an L_C_ preferred extension. Quan et al. [56] analysed different graphite samples from different mining sites and reported L_C_ values between 10 and 18 nm and L_A_ values between 22 and 40 nm. For the L_C_ stacking height, however, they reported values between 10 and 18 nm, which were four to five times lower than those calculated in this investigation, while reported L_A_ values were in good agreement with our calculations.

After synthesis and carbonization, the yielded carbons showed differences in density, with the highest for CXG_50_ and the lowest for CXG_20,000_. CXG_50_ especially formed very hard and dense particles. One possible explanation might be the higher concentration of sodium carbonate in the range of R/C_50–1500_ that favoured fast formation of big aggregates [39]. Immediately after adding formaldehyde to the R/C_50_ solution, the colour started to change from clear white to red/orange. After 2 h the polymerization was completed, resulting in a dark red colour. For R/C_500–1500_ the whole polymerization was finished after 18–24 h. For >R/C_1500_ the polymerization took 35–45 h due to a slow reaction rate. It appears that a slow polymerization reaction rate favoured the formation of many small aggregates which resulted after carbonization in less dense carbon particles. At very low Na_2_CO_3_ concentrations, as in the R/C_10,000_ and R/C_20,000_ synthesis mixtures, the solution was colourless even after 60 h reaction time.

### 3.3. Raman

**Figure 4:**
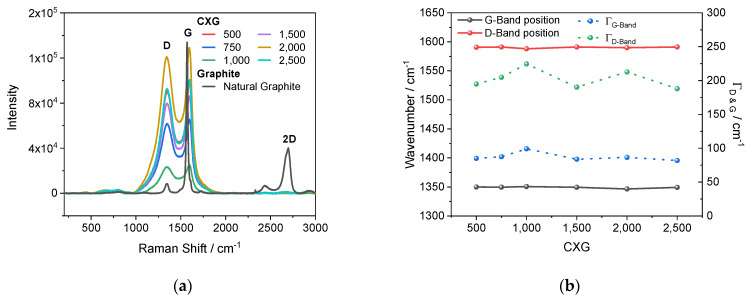
Raman spectra of selected CXG (**a**). Position of D and G-band as well as Γ_D-_ and Γ_G-band_ as a function of R/C value (**b**).

The Raman spectra of the CXG samples (see Figure 4a) showed a clear D- and G-band at 1346 and 1595 cm^−1^ which were comparable to those for CXGs synthesized by Kakunuri et al. [47]. An intense D-band mainly arises from the polymerization reaction products of resorcinol and formaldehyde coming from numerous sp^3^ orbitals of –CH_3_, –OH and other functional groups which result after carbonization in the formation of carbon domains with multiple defect structures [48,57]. The assignment of D- and G-bands in graphitic structures, such as natural graphite, is straightforward. The G-band around 1575 cm^−1^ arises from the E_2g_ mode of all present sp²-hybridized carbons located in-plane in the carbon chains and rings and can be visualized as bond-stretching, whereas the D-band in graphitic structures can be explained by a “breathing” of the A_1g_ mode of the carbon chains and rings [58]. Therefore, the observed G-band of the CXGs can be explained by bond-stretching of carbon rings. Natural graphite has a true second-order 2D-Band at 2750 cm^−1^ coming from overtones and disorder induced by in-plane modes [59,60,61,62].

The intensity of a Raman signal is directly linked to the density of the material and the fourth power of the excitation frequency. Hence, since excitation frequency was kept constant over the time, sample density played a dominant role. Qualitatively, the strongest Raman intensity was obtained with the CXG_2000_ sample, followed by the CXG_2500_, CXG_500_ and CXG_1500_ samples. The intensities of the G-band assigned peak surpassed those of the D-band signals in all measurements. To obtain more information about carbon composition regarding the graphitic and non-graphitic domains, the I_D_/I_G_ peak intensity and corresponding A_D_/A_G_ peak surface ratio were calculated. For samples with low disorder level, the calculation of A_D_/A_G_ ratio is more straightforward because it represents the probability of the whole phonon-coupling process including uncertainty [63,64,65]. For samples with high disorder level the decoupled full-width at half maximum (FWHM) values of the D-band and G-band (Γ_D-Band_ and Γ_G-Band_/cm^−1^) provide more information.

Structural properties of graphitic and non-graphitic carbons, and the correlation of Γ_D-Band_ and Γ_G-Band_ with the lateral crystal size, have been reported by several groups [64,66,67]. Schuepfer et al. [48] demonstrated that increase in carbonization temperature up to 3000 °C strongly improved the graphitization level, especially in pitch-based samples, whereas the D-band was still visible for resin-based carbons even at this high temperature level. The authors suggested that a greater Γ_G-Band_ value is correlated with smaller lateral crystallite sizes. We observed a median Γ_D-Band_ ranging from 224–187 cm^−1^, while for Γ_G-Band_, a median value of 86 cm^−1^ was recorded (Figure 4b). The highest Γ_D-Band_ of 224 cm^−1^, as well as a Γ_G-Band_ of 99 cm^−1^, were measured for CXG_1000_, while the smallest Γ_G_ and Γ_D-Band_ values were calculated for CXG_2500_ at only 82 cm^−1^ and 188 cm^−1^, respectively. It was found that the measured Γ_D-Band_, as well as the Γ_G-Band_, followed a similar trend. The calculated Γ_G-Band_ values were slightly greater than those from Schuepfer et al. [48], ranging from 40–80 cm^−1^ for L_A_ > 2 nm. It should be noted that the as-synthesized resins were not identical. Similar Γ_G-Band_ values around 80 cm^−1^ were reported for graphene by Cancado et al. [68]. The corresponding L_A_ values indicated sizes < 1–2 nm which fits well with the XRD results of this investigation.

The measured Γ_D_ values > 200 cm^−1^ indicate L_D_ values ≈ 2–1 nm compared with data from Ferreira et al. [64]. Since the crystallite size of our as-prepared carbon xerogels was quite similar, accurate differentiation between punctual and edge defects, as well as L_D_ from L_A_ values, was not possible.

### 3.4. Electronic Conductivity

**Figure 5:**
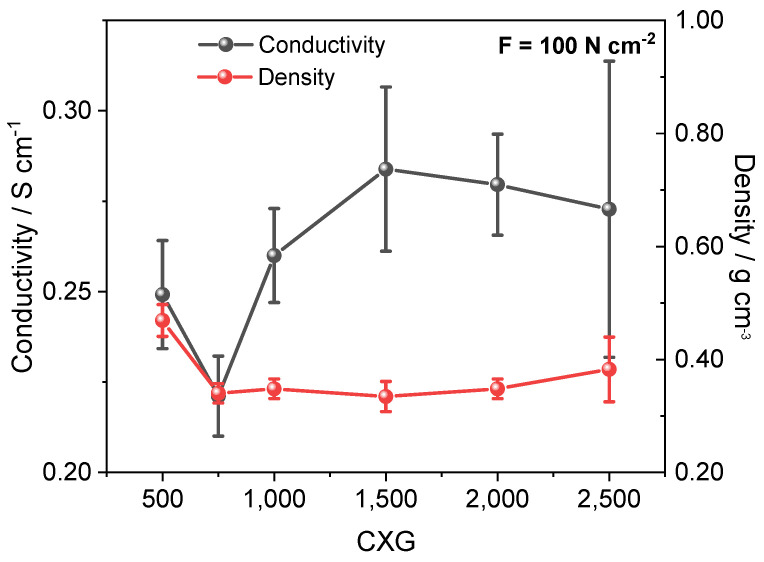
Conductivity and density values of CXG powders as a function of R/C ratio.

Electronic conductivity and density values of as-prepared carbon xerogels plotted in Figure 5 followed a similar trend. The conductivity increased from 0.249 ± 0.015 S cm^−1^ in CXG_500_ to 0.284 ± 0.023 S cm^−1^ in CXG_1500._ The lowest conductivity was measured for CXG_750_ with 0.221 ± 0.011 S cm^−1^. The overall measured conductivity was around 0.260 S cm^−1^ which was consistent with xerogel values reported by Tondi et al. [69] (1.13–1.42 S cm^−1^), while the calculated density was relatively constant around 0.40 g cm^−3^. It was observed that the conductivity fluctuated in close accordance with the Γ_D_ and Γ_G-Band_ values, as calculated from Raman measurements, that can be assigned to the particle size L_A_ and point-defect distance L_D_. As the average Γ_G_ was nearly constant over all CXG ratios (≈86 cm^−1^), and the measured conductivity fluctuated with little variation between 0.221 and 0.284 S cm^−1^, we conclude that the through-plane conductivity of carbon xerogels was mainly influenced by sp²-hybridized carbons, while small changes in conductivity were a result of changes in L_A_ and L_D_. For comparison, the natural graphite exhibited a conductivity of 19.3 S cm^−1^ which was nearly two magnitudes higher, but only 5–10% of published values for other natural graphites (152–223 S cm^−1^) [70,71]. These deviations can be explained by the difference in the analytical method used, and even more by the analysed specimen properties (e.g., type, shape, origin). Marinho et al. [72] analysed different types of graphite, graphene and multi-wall carbon nanotubes (MWCNT) at a pressure of 500 N cm^−2^, which was five times higher than the pressure applied in this investigation, and they found a bulk conductivity of 21.2 S cm^−1^ that was comparable with our results. A detailed overview is given in Table A1.

### 3.5. SEM

**Figure 6:**
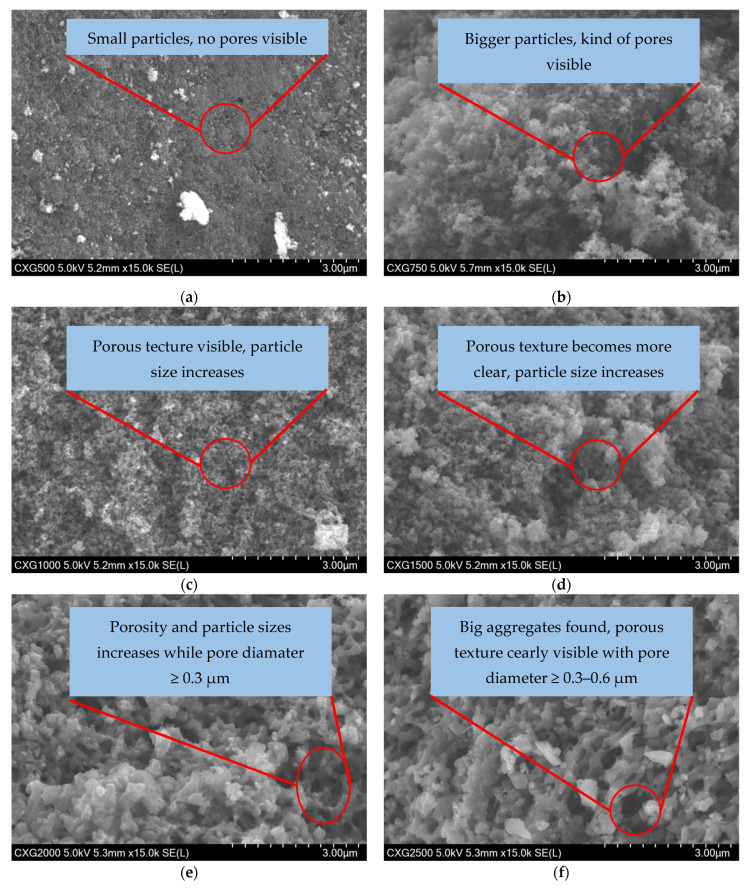

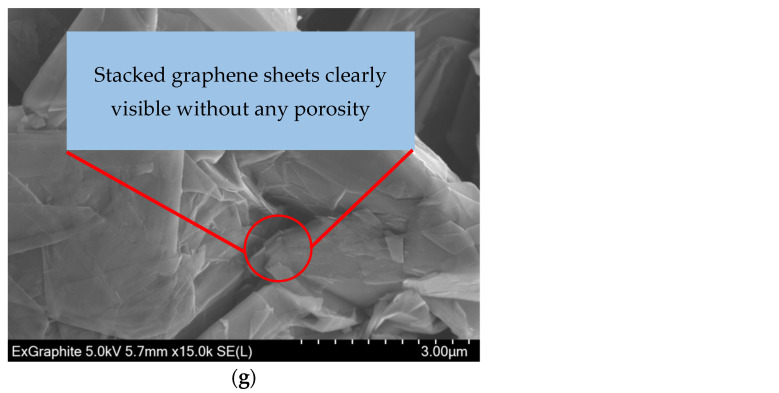
SEM images of CXG_500_ (**a**), CXG_750_ (**b**), CXG_1000_ (**c**), CXG_1500_ (**d**), CXG_2000_ (**e**), CXG_2500_ (**f**) and natural graphite powders (**g**) at 5 kV.

Figure 6 shows SEM images of CXG_500–2500_ at 5 kV acceleration voltage and 15 k magnification. The mean size of carbon xerogel particles clearly increases with increasing R/C values from 0.079 µm in CXG_500_ to 0.804 µm in CXG_2500_. Similar surface textures have been reported elsewhere for comparable carbon xerogels [31,73,74]. In contrast, natural graphite particles consist of very wide stacked two-dimensional platelets with geometric shape >1 µm.

### 3.6. Half-Cell Experiments

**Figure 7:**
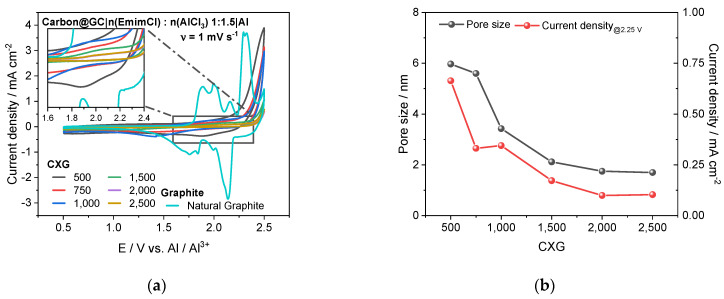

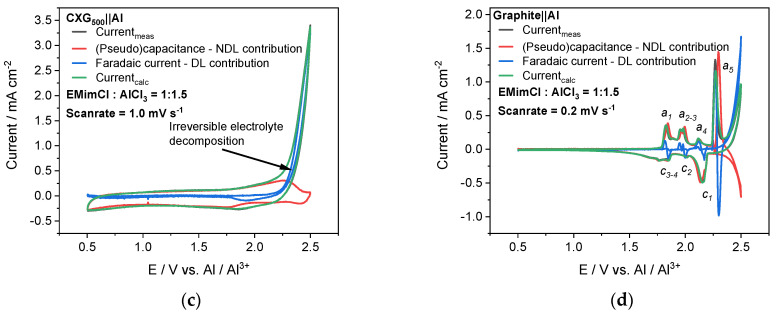
CVs of selected CXGs and natural graphite in 1:1.5 n(EmimCl):n(AlCl_3_) electrolyte (**a**) and influence of average pore size on max. current density taken from CVs of selected CXG at 2.25V (**b**). Deconvoluted current contribution of non-diffusion limited (pseudo)capacitance and diffusion-limited Faradaic currents in CXG_500_||Al (**c**) and in graphite||Al (**d**). “(pseudo)capacitance” designation comprises both EDLC and pseudocapacitance contribution.

With the exception of CXG_500_ and CXG_750_ behaviour, the cyclic voltammograms of most CXG carbons in 1:1.5 EMiMCl:AlCl_3_ shown in Figure 7a do not exhibit any pronounced redox peaks in comparison to those observed for natural graphite material. The CV of mesoporous carbon CXG_500_ reached a maximum current density of 0.7 mA cm^−2^ at 2.25 V that might be assigned to the intercalation step of AlCl_4_^−^ ions into the carbon matrix shortly before the onset potential of electrolyte decomposition. The relatively small but broad reduction peak at 1.85 V (inset in Figure 7a) might be attributed to a sluggish de-intercalation process. In the CV of CXG_750_, a slightly decreased maximum current density of 0.3 mA cm^−2^ at 2.25 V and a small reduction peak around 1.4 V can be seen. As shown in Figure 7b, the onset potential of the presumed AlCl_4_^−^ intercalation-related step continuously increased with increasing R/C ratio as mean pore size decreased. The CV of CXG_1000_ exhibited a weakly pronounced reduction peak at 1.2–1.3 V. The maximum current density produced by CXG_1500–2500_ samples measured at 2.25 V decreased from 0.17 mA cm^−2^ for CXG_1500_ to 0.10 mA cm^−2^ for CXG_2500_, indicating poor accessibility of active sites due to the predominantly microporous structure, as explained in Section 3.1. In contrast, the cyclic voltammograms of natural graphite (Figure 7a) show five distinct oxidation peaks during the oxidation step, where every peak corresponds to an intercalation step of chloroaluminate anions into graphene sheets. The first peak at 1.8 V can be related to a *stage-6* intercalation in which in every sixth graphene sheet a chloroaluminate anion was intercalated. The assignment of the peaks is then straightforward: the main-peak at 2.3 V corresponds to *stage-3* intercalation in which at every third graphene layer a chloroaluminate anion was inserted [75,76,77]. This stage also represents a fully charged graphite AIB. The peak at 2.5 V might be an overlapping of the AlCl_4_^−^ intercalation step with the onset of electrolyte oxidation. In the cathodic scan, corresponding reduction peaks related to the reversible deintercalation process are visible. Similar CVs of graphite in AIB cells have been reported elsewhere [6,78,79], though some divergences with respect to peak number and peak location may exist depending on the graphite sample used and the applied potential window.

To determine the contribution of (pseudo)capacitance and the Faradaic contribution to overall current, CVs were carried out with CXG_500_ and graphite materials at different scanning rates (see Figure A2a,b) and evaluated using Dunn’s method where the “(pseudo)capacitance, (Q_(Pseudo)cap_)” designation implies here both an electrochemical double-layer capacitance EDLC (Q_EDLC_) and a pseudocapacitive (Q_Pseudocap_) contribution. The resulting deconvoluted CVs are presented in Figure 7c,d. In the potential region of 0.5–2.0 V, the CV of CXG_500_ shows a broad current vs. voltage profile in comparison to graphite which can be attributed to a Q_(Pseudo)cap_ value of 475 mC. The contributions from the Q_EDLC_ and Q_Pseudocap_ were determined to be 445 mC (94%) and 30 mC (6%), respectively. The latter can be assigned to insertion of chloroaluminate into the carbon host matrix. This observation is supported by the b-value of 0.86 at 2.25 V determined by Lindströms method [80] which indicates a mixed regime of fast non-diffusion limited (b-value = 1.0) and diffusion-limited (b-value = 0.5) reactions which is typical for intercalation reactions [81]. At potentials between 1.0 V and 2.0 V, the b-value was in the range of 0.97–1.01, as expected from the EDLC contribution. The plots and detailed results for determination of the b-values are shown in Figure A3a,b and Table A3. At higher anodic potentials than 2.25 V, the diffusion-limited (DL) Faradaic currents rise exponentially up to 3.4 mA cm^−2^ due to additional irreversible electrolyte decomposition. In the cathodic scan, a diffusion-limited reaction was observed between 2.1 V and 1.8 V and assigned to the deintercalation of chloroaluminate. The discrepancy of measured currents (black line) and experimental calculated current (green line) was less than 5%. In Figure 7d the disentangled current contributions are shown for the natural graphite. The evaluation of the data was even more complex, since the intercalation peaks drift with scan-rate. Therefore, we acquired CV scans with very low scan rates at 0.1–0.5 mV s^−1^ to ensure a minimal impact of the peak drift. The data shows that the measured charge was dominated by pseudocapacitive contributions. The b-values (see Figure A3c,d and Table A4) of the anodic peaks had values between 0.89 (*a*_1_) and 0.67 (*a*_5_) for the main intercalation peak at 2.29–2.27 V. This indicates, similarly to CXG_500_, a mixed regime of non-diffusion and diffusion limited currents, whereas for the main intercalation peak, the diffusion limitation dominates. This is supported by the deconvoluted current contributions, where, for each intercalation peak, diffusion-limited Faradaic currents are found, most intensely around the main intercalation peak. The mismatch of the measured and disentangled currents is caused by the peak drift, which was around 27 mV around the main intercalation peak. The cathodic b-values were around 0.91–0.97 which indicates nearly pure pseudocapacitive behaviour. The contribution of the EDLC to the overall charge (0.5–2.5V) was 97.6 mC (8.4%), since the surface area of the graphite was very small compared to that of carbon xerogels. The contribution of the pseudocapacitance and the Faradaic currents could not be calculated due to the error in peak shift. The data acquisition and evaluation need to be optimised. For AIBs, typical values for the contribution of pseudocapacitance, inclusive of EDLC, are around 70–80%, while for Faradaic currents, values of 30–20% have been reported [81].

### 3.7. Cycling Performance of AIB Cells

**Figure 8:**
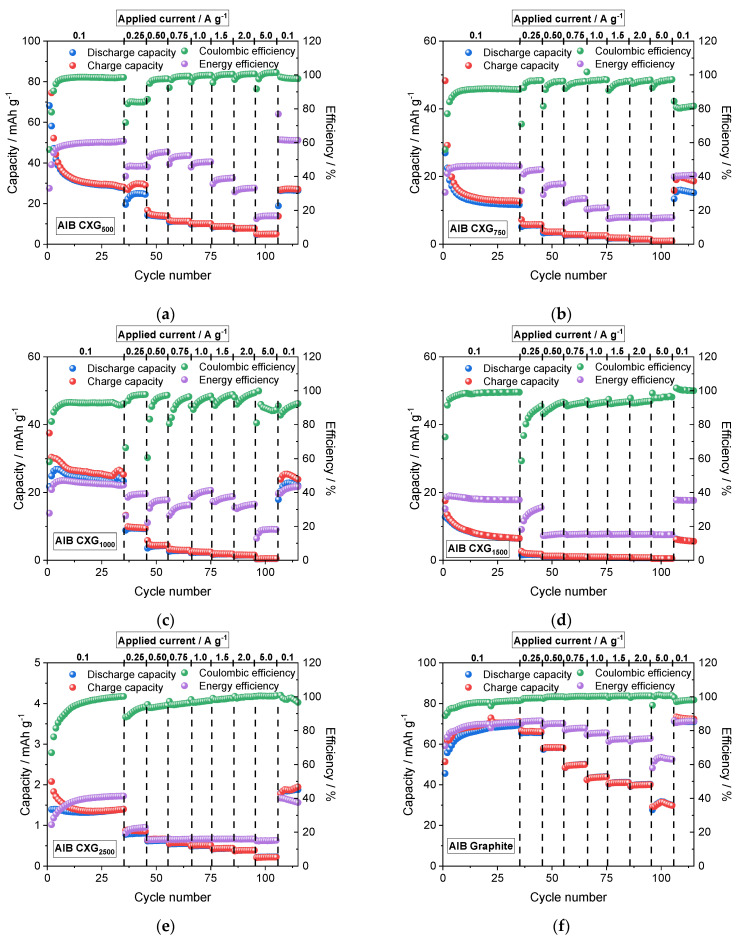
Charging/discharging experiments of CXG_500_ (**a**), CXG_750_ (**b**), CXG_1000_ (**c**), CXG_1500_ (**d**), CXG_2500_ (**e**) and natural graphite (**f**) in Swagelok-type straight-cells with tungsten as cathodic current collector and aluminium rod as anode in n(EMimCl):n(AlCl_3_) 1:1.5 electrolyte.

**Figure A1:**
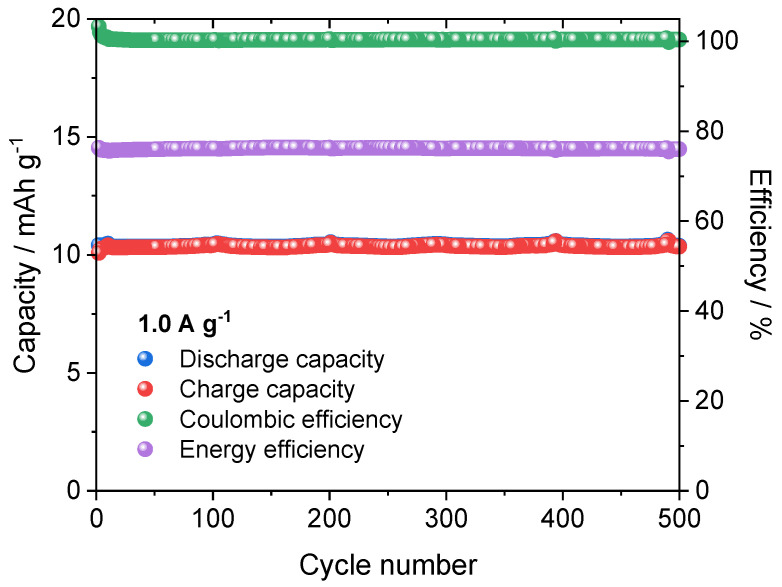
Long-term cycling of CXG_500_ in AIB full-cell setup.

**Figure A2:**
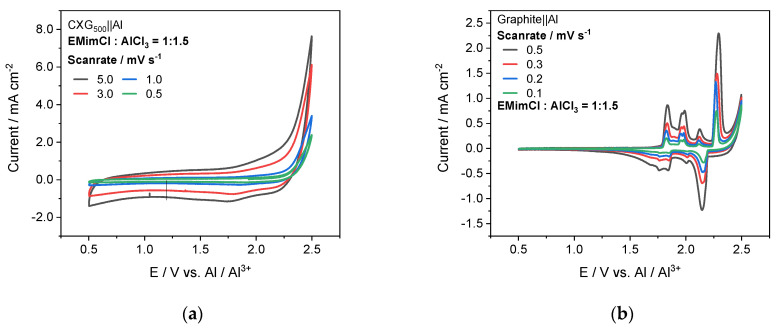
Scan-rate variation of cyclic voltammograms for CXG_500_||Al (**a**) and natural graphite||Al (**b**).

**Figure A3:**
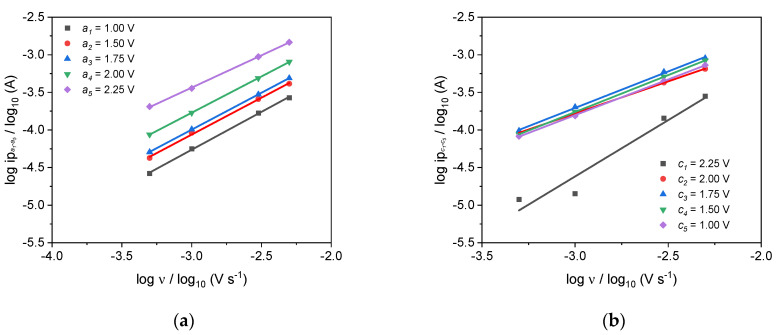

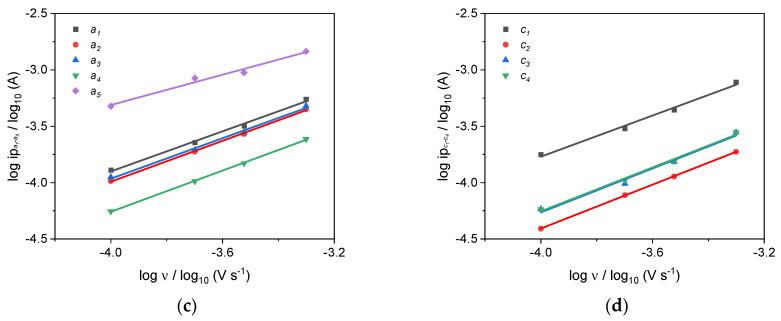
Determination of b-values in CXG_500_||Al at the anodic (**a**) and cathodic (**b**) CV scan and for natural graphite for intercalation peaks a_1–5_ (**c**) and deintercalation peaks c_1–4_ (**d**).

The most promising carbon xerogels were further tested in the AIB cell with regards to their capacity behaviour as a function of applied current density up to 5.0 A g^−1^ and compared with the performance of a cell with natural graphite. As expected from half-cell experiments, AIB with CXG_500_ material exhibited best charge/discharge behaviour up to 28 mAh g^−1^ at 0.1 A g^−1^ with nearly 98% coulombic and 60% energy efficiency (see Figure 8). This value is in good agreement with recently published values for soft-carbon (SC) electrodes from Li et al. [82] who found a capacity of 28.2 mAh g^−1^ at 0.3 A g^−1^ current density. With increasing R/C ratio, however, the performance of AIB dropped continuously from about 15–20 mAh g^−1^ for cells with CXG_750_ and CXG_1000_ materials to a few mAh for the other systems. This can be ascribed to a decrease in average pore size that seems to hinder active species diffusion to intercalation sites within the carbon matrix. In comparison, the reversible capacity of the AIB cell with a natural graphite cathode reached 69 mAh g^−1^. At a maximum current density of 5 A g^−1^, the calculated specific capacity of AIB with CXG_500_ amounted to 10 mAh g^−1^, one third that measured for the AIB cell with a natural graphite electrode (30 mAh g^−1^). In long-term cycling at 1.0 A g^−1^, the capacity retention over 500 cycles was around 100%, with a specific measured capacity of 10–11 mAh g^−1^ (see Figure A1 in Appendix A). Moreover, during the first 35 cycles @ 0.1 A g^−1^, the capacity of all AIBs with carbon xerogels diminished substantially, whereas the capacity of AIBs with graphite material increased. The change in capacity during the initial stage might be attributed to the irreversible intercalation of some AlCl_4_^−^ anions in the carbon structure [83] leading to a re-arrangement of the host matrix, with either capacity decrease, in the case of cells with CXG, or capacity increase (activation phase), in the case of AIBs with NG material. The activation of AIBs with natural graphite is not fully understood and is the subject of many investigations [5,77,83,84].

**Figure 9:**
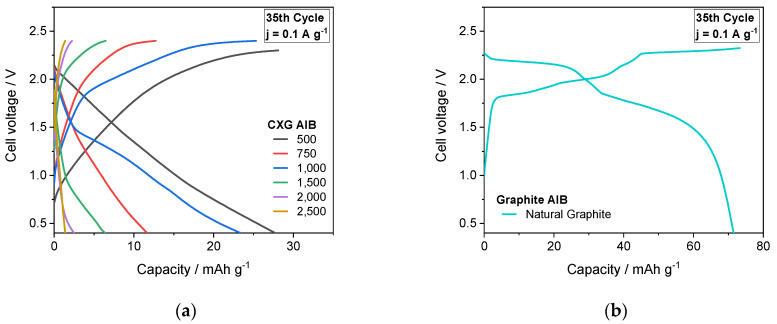
Charging and discharging curves of AIBs equipped with CXG_500–2500_ (**a**) and natural graphite for comparison (**b**).

The 35th charge/discharge cycle carried out at 0.1 A g^−1^ of the different AIB cells is shown in Figure 9. Among the carbon xerogel materials, AIB with CXG_500_ showed a discharge capacity of 28 mAh g^−1^ at 0.1 A g^−1^ that can be attributed to its high mesoporous volume. With decrease in V_mes_, the discharge capacity of AIB faded to 12–23.3 mAh g^−1^ in AIBs with CXG_750_ and CXG_1000_, and even lower for materials with R/C values > 1500 (7–1 mAh g^−1^). The discharge capacity of AIB with NG amounted to 69 mA g^−1^. With the exception of AIBs with CXG_1000_ material, the cell voltage decreased/increased continuously from open-circuit down to the cut-off voltage in contrast to the polarization curves of AIBs with NG, in which distinct plateaus were visible. This correlates well with the cyclic voltammograms shown in Figure 7 and is an additional indication of different intercalation/deintercalation mechanisms of AlCl_4_^−^ ions due to the differences in gallery/crystallite length (2 nm for CXG vs. 30 nm for NG). Since aluminium chloride is not involved in any redox process (only 3+ valence), the strong Lewis acid is assumed to coordinate with non-bonded electron pair donors [85].

The observed plateaus are related to different staging of chloroaluminate intercalation compounds into the graphite matrix [75,77]. The main plateau at 2.25 V is assigned to the *stage-3* intercalation and indicates a fully charged AIB in which only one of three graphene layers is filled with chloroaluminate anions and the two intermediate layers are completely empty [75].

**Figure 10:**
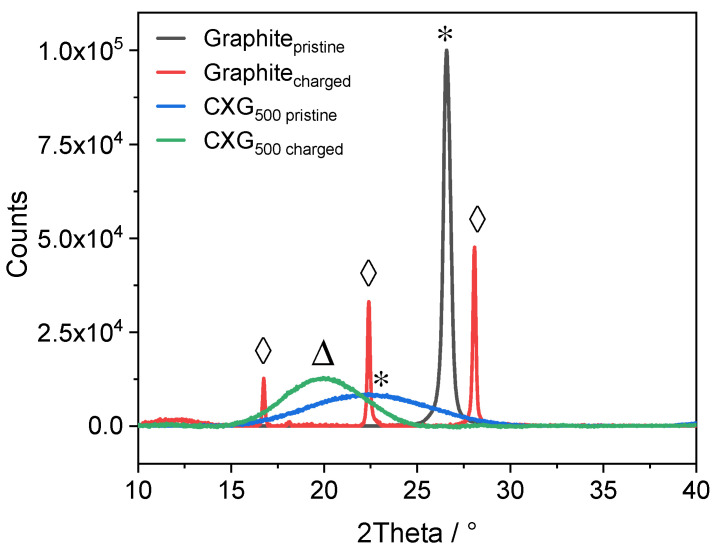
Ex situ XRD analysis of pristine and fully charged natural graphite and CXG_500_ electrodes. Peaks marked with (*) indicate position of (002) peak in both pristine electrodes. The peak marked with (∆) is assumed to be associated with the (002) peak shift due to intercalated species in CXG_500_. Peaks indexed with (◊) are related to GIC-induced peaks in natural graphite sample.

An ex situ XRD analysis of a fully charged natural graphite and CXG_500_ electrode is shown in Figure 10 and compared to that of pristine samples. The (002) peak at 26.5° in pristine graphite vanished completely, while in the charged graphite electrode, three new peaks at 16.7° (d-spacing 5.31 Å), 22.4° (d-spacing 3.98 Å) and 28.0° (d-spacing 3.18 Å) appeared which resulted from coordination of the intercalated chloroaluminate anions with delocalized π-electrons of the sp^2^ orbitals from the graphite material building, forming so-called graphite intercalation compounds (GIC), as suggested in [11,75,83]. The assignment of the GIC peaks in (00l) nomenclature is straightforward: the most dominant peak is labelled as (00n+1), while the second most dominant peak is (00n+2), where (n) represents the number of a n-staged GIC. The correlation of the periodic distance (I_C_) of graphene layers, the intercalant gallery height (d_i_), the gallery expansion ∆d and the intercalation stage (n) are given by the following equation [11,83,86,87,88]:

(2) $$I_{C}=d_{i}+3.35\;Å\cdot \left(n-1\right)=\Delta d+\left(3.35\;Å\cdot n\right)=l\cdot d_{\mathrm{obs}}$$

The distance 3.35 Å is given for an ideal graphite. The (l) and (d_obs_) are the (00l) reflex and d-spacing of the observed GIC peaks, respectively. The intercalation stage (n) can be obtained by comparing the experimental d_(n+2)_/d_(n+1)_ ratios with standard values, as listed in Table 1.

The experimentally obtained d-spacing of the observed GIC can then be converted into an intercalation stage number n. For the peak pair (1) at 2θ _(00n+1)_ = 28.0° (d-spacing 3.18 Å) and 2θ _(00n+2)_ = 22.4° (d-spacing 3.98 Å), the ratio d_(n+2)_/d_(n+1)_ was determined to be 1.25 which can be associated with a *stage-3* GIC. For the peak pair (2) at 2θ _(00n+1)_ = 22.4° and 2θ _(00n+2)_ = 16.7° (d-spacing 5.31 Å), the ratio d_(n+2)_/d_(n+1)_ was calculated to be 1.33 which was assigned to a *stage-2* GIC.

Inserting experimental values in equation (2), the periodic distance I_C_ was calculated as 15.93 Å. The intercalant gallery height (d_i_) for the *stage-2* and *stage-3* GIC were consequently determined to be 12.58 Å and 9.23 Å, respectively. The results for the gallery expansion (∆d), which were directly linked to the intercalant species, was 9.23 Å for *stage-2* GIC and 5.88 Å for the *stage-3* GIC. Since the radius of the intercalated AlCl_4_^−^ was around 5.28 Å [23], the gallery expansion in *stage-3* is quite compatible with the AlCl_4_^−^ radii. The enormous expansion in *stage-2* might suggest ongoing delamination of the graphene sheets. Another possibility might be the intercalation of larger Al_2_Cl_7_^−^ anions which have twice the size of AlCl_4_^−^ and can therefore be responsible for the extremely large expansion/delamination. An overview of our results, as well as some from the literature, is given in Table 2. It seems that there is a possible confusion between (d_i_) and (∆d) in [11], because the published value for (di) differs too much between our results and already published values for the same dominant stage. The claimed (di) seems to be more logical if denoted (∆d).

According to some literature sources, however, *stage-2* intercalation should happen only at voltages higher than 2.6 V [75]. The observed gallery expansion (∆d) value is comparable with those from Elia et al. [83], where small discrepancies can be a result of the difference in dominant stage. Additionally, it should be noted that numerous graphite products/grades are available on the market that make accurate comparison of studies difficult. The identification of GICs in AIB graphite layers is still a challenge. By evaluating C K-edge XAS spectra of different charged graphite electrodes, Wang et al. [89] postulated that the peak at 287.3 eV results from formation of chemical Cl-C bonds after the AlCl_4_^−^ intercalation step.

In contrast to graphite, the mechanisms of the intercalation process of AlCl_4_^−^ species in CXGs have not yet been studied in detail. Since CXGs do not have a well-ordered crystal structure, the diffractogram did not show any sharp GIC peaks as in graphite. Nevertheless, the main peak at 2θ = 22.27° (d-spacing 3.98 Å) of pristine CXG_500_ shifted to 2θ = 19.73° (d-spacing 4.50 Å) in a charged state. This shift can be interpreted as a result of AlCl_4_^−^ intercalation into the carbon matrix since CXGs also have delocalized π-electrons from the sp2 orbitals of the aromatic resorcinol ring which are responsible for pseudo-capacitive behaviour during the intercalation step. This assumption should be confirmed by XPS analysis.

## 4. Conclusions

In this study, the feasibility of reversible chloroaluminate anion intercalation into CXG material during AIB cycling was investigated. The most relevant results of the material texture and electrochemical charge/discharge experiments are summarized in Table 3.

Synthesis of CXG was carried out using a template-free route with resorcinol as the carbon source and formaldehyde, followed by a carbonization step at 800 °C in N_2_ atmosphere. The influence of the resorcinol to NaCO_3_ catalyst ratio in the range of 500 < R/C < 20,000 during synthesis on CXG particle morphology and electrochemical performance was studied. Physisorption analysis of carbon powder materials revealed that CXG_500_ had the highest mesopore volume (~77%) and pore size (6 nm) that continuously decreased with higher R/C ratios. For R/C > 2500 samples, micropore domains clearly predominated (>90%). The surface area of the prepared CXG was around 950 m^2^ g^−1^, with a maximum of 1000 m^2^ g^−1^ for CXG_500_.

The peak positions of the D-band and G-band in Raman spectra remained unchanged at 1346 cm^−1^ and 1595 cm^−1^, respectively, for all synthesized CXG ratios, indicating similar carbon materials. A detailed analysis of Raman spectra revealed that the synthesized CXG particles had crystallite dimensions smaller than 2 nm. Variations in Γ_D-Band_ from 224 cm^−1^ (CXG_1000_) to 188 cm^−1^ (CXG_2500_) were assigned to fluctuations in L_A_, together with point-to-point defects L_D_. The Γ_G-Band_ was almost constant at 86 cm^−1^ with 99 cm^−1^ (CXG_1000_) and 82 cm^−1^ (CXG_2500_).

Through-plane conductivity measurements of CXG_500–2500_ showed conductivity lying between 0.25–0.28 S cm^−1^. Variations in conductivities with respect to the CXG ratio could be ascribed to fluctuations of Γ_D-Band_ while the Γ_G-Band_ was understood to be beneficial for conductivity.

Crystallographic analysis of CXG showed that the stacking width L_A_ (1.39–1.57 nm) and stacking height L_C_ (0.94–1.06 nm) were almost independent of catalyst concentration.

The highest discharge capacity of 24–29 mAh g^−1^ in AIB cells was observed for CXG_500_ material, compared to 69–73 mAh g^−1^ for AIB with a graphite electrode. With higher CXG ratios, the discharge capacity was significantly reduced compared to CXG_500_ due to lower porosity.

Ex situ XRD analysis of a charged CXG electrode exhibited a small peak shift of the main peak from pristine 2θ = 22.27 of about 2.54° towards smaller degree that might be an indication of intercalation of chloroaluminate into the carbon structure. For the charged graphite electrode, the dominant intercalation steps were determined to be *stage-2* and *stage-3*, with gallery expansion of the adjacent graphene layers of about 5.88 Å and 9.23 Å, respectively.

In this study, we have demonstrated for the first time the feasibility of carbon xerogels prepared by a simple and cheap soft-template route as positive electrode material for aluminium ion batteries, paving the way for future investigations. Further improvements are still needed to enhance the overall capacity of CXGs, and, more particularly, the contribution of AlCl_4_^−^ ion intercalation (pseudocapacitance) to overall capacity that might be achieved by enhancing the graphitization level of CXG through, for example, graphene doping. 

## Figures and Tables

**Table 1:** Correlation of intercalation stage (n) and theoretical d_(n+2)_/d_(n+1)_ ratio according to [83,87].

Stage (n)	d_(n+2)_/d_(n+1)_ Ratio	Dominant (00l) Peak
1	1.50	002
2	1.33	003
3	1.25	004
4	1.20	005
5	1.17	006
6	1.14	007

**Table 2:** Dominant stage and calculated gallery height and expansion from ex situ XRD measurements of charged graphite electrode.

	Peak Pair (1)	Peak Pair (2)
2θ (00n+1)/2θ degree	28.0	22.4
2θ (00n+2)/2θ degree	22.4	16.7
d_obs(n+1)_/Å	3.18	3.98
d_obs(n+2)_/Å	3.98	5.31
d_obs(n+2)_/d_obs(n+1)_	1.25	1.33
Dominant stage (n)	3	2
Periodic distance I_C_/Å	15.93	
Al_x_Cl_y_ gallery height (d_i_)/Å	9.23	12.58
Al_x_Cl_y_ gallery expansion (∆d)/Å	5.88	9.23
Literature for (d_i_)/Å (dominant stage)	5.7 (4) [11] 9.54 (3) [75] 9.59 (4) [83]	
Literature for (∆d)/Å (dominant stage)	6.24 (4) [83]	

**Table 3:** Summary of relevant textural properties of synthesized CXGs and natural graphite tested in AIB cell with corresponding performance values at 0.1 A g^−1^.

CXG	Spec. Area m² g^−1^	V_Me_ cm^3^ g^−1^	Mean Pore Size nm	L_A_/L_C_ nm	Γ_D-Band_ m^−1^	Conduct. S cm^−1^	Q_dis_ @ 0.1 A g^−1^ 30th Cycle mAh g^−1^	CE%	EE %
500	1004	0.77	6.0	1.57/0.96	195	0.249	28.7	98.2	60.2
750	919	0.57	5.6	1.45/0.99	205	0.221	11.8	91.5	46.1
1000	894	0.26	3.4	1.45/1.04	224	0.260	23.3.	93.0	44.8
1500	922	0.08	2.1	1.39/1.06	190	0.284	6.6	99.1	35.7
2500	944	0.02	1.7	1.45/0.94	188	0.273	1.4	99.7	41.1
NG	22	0.03	13.0	9.0–41.0	110	19.3	68.8	97.4	84.7

## Appendix A

**Table A1:** Electronic conductivity values of carbon xerogels and natural graphite.

CXG	Resistance Ω	Spec. Resistance Ω cm	Conductivity S cm^−1^	Density g cm^−3^
50	0.400	2.565	0.390	0.781
500	1.040	4.012	0.249	0.469
750	1.573	4.522	0.221	0.340
1000	1.385	3.846	0.260	0.348
1500	1.252	3.522	0.284	0.334
2000	1.221	3.577	0.280	0.348
2500	1.186	3.665	0.273	0.382
3000	0.900	3.031	0.330	0.402
3500	1.124	3.640	0.275	0.403
4000	0.958	3.885	0.257	0.515
4500	0.905	4.044	0.247	0.555
5000	1.045	4.109	0.243	0.491
10,000	0.905	4.718	0.212	0.628
20,000	0.623	3.551	0.282	0.693
NG	0.017	0.052	19.322	0.782

**Table A2:** Summary of physisorption measurements of carbon xerogels and natural graphite.

CXG	Area_DFT_ m^2^ g^−1^	Fit Error %	A_mic_ (DR) m^2^ g^−1^	A_mes_ m^2^ g^−1^	V_sum_ cm^3^ g^−1^	V_mic_ (DR) %	V_mes_ %	Pore Size nm
500	1004	0.418	691	312	1.00	23.1	76.9	6.0
750	919	0.570	778	141	0.83	31.4	68.6	5.6
1000	894	0.194	717	177	0.50	47.8	52.2	3.4
1500	922	0.039	736	187	0.33	75.6	24.4	2.1
2000	918	0.037	716	202	0.27	88.5	11.5	1.7
2500	940	0.019	722	218	0.27	90.8	9.2	1.7
3000	948	0.037	763	184	0.27	95.5	4.5	1.7
3500	975	0.022	739	237	0.25	97.2	2.8	1.6
4000	915	0.131	726	189	0.25	95.3	4.7	1.6
4500	931	0.073	701	230	0.24	99.2	0.8	1.6
5000	934	0.077	717	217	0.24	98.4	1.6	1.6
10,000	926	0.217	724	201	0.25	98.0	2.0	1.6
20,000	911	0.017	689	222	0.25	92.7	7.3	1.6
NG	22	1.298	/	22	0.07	57.1	42.9	13.0

The peaks named *a*_1_–*a*_5_, as well as *c*_1_–*c*_5_ in CXG_500_ CVs, were randomly selected to give a good overview over the whole CV. Please note that the nomenclature has nothing to do with the true intercalation or deintercalation peaks with the same nomenclature as observed with natural graphite.

**Table A3:** Overview of results of CXG_500_||Al experiments reported in Figure A2a.

**b-Value** **(Lindström’s Method)**	**Anodic**	***a*** **_1_**	***a*** **_2_**	***a*** **_3_**	***a*** **_4_**	***a*** **_5_**
		1.01	0.98	0.98	0.97	0.86
	**Cathodic**	***c*_1_**	***c*_2_**	***c*_3_**	***c*_4_**	***c*_5_**
		1.50	0.85	0.97	0.98	0.95
**Charge** **mC**		**Rel. Charge Ratio** **%**				
**Q_Measured_**	968	-				
**Q_Calculated_** *	1019	100				
**Q_Farad_**	544	**Q_Farad_/Q_Calculated_**		53		
**Q_(Pseudo)cap_**	475	**Q_(Pseudo)cap_/Q_Calculated_**		47		
**Q_EDLC_**	445	**Q_EDLC_/Q_(Pseudo)cap_**		94		
**Q_Pseudocap_**	30	**Q_Pseudocap_/Q_(Pseudo)cap_**		6		
**Error (measured vs. calculated charge/%)**		5				

* Q_Calculated_ = Q_Farad +_ Q_(Pseudo)capacitance_ with Q_(Pseudo)capacitance_ = Q_EDLC_ + Q_Pseudocapacitance._

**Table A4:** Overview of results of graphite||Al experiments reported in Figure A2b.

**b-Value** **(Lindström’s Method)**	**Anodic**	***a*** **_1_**	***a*** **_2_**	***a*** **_3_**	***a*** **_4_**	***a*** **_5_**
		0.89	0.91	0.90	0.91	0.67
	**Cathodic**	***c*_1_**	***c*_2_**	***c*_3_**	***c*_4_**	***c*_5_**
		0.91	0.97	0.97	0.97	0.97
**Charge** **mC**		**Rel. Charge Ratio** **%**				
**Q_Measured_**	1110	100				
**Q_EDLC_**	97	**Q_EDLC_/Q_Measured_**		8		

Since we were not able to calculate the separate contribution of (pseudo)capacitance and Faradaic currents to overall charge value, Q_EDLC_ ratio is referred to as Q_Measured_.
